# Maximizing dietary information retrievable from carcasses of Great Cormorants *Phalacrocorax carbo* using a combined morphological and molecular analytical approach

**DOI:** 10.1111/ibi.12337

**Published:** 2015-12-15

**Authors:** Johannes Oehm, Bettina Thalinger, Hannes Mayr, Michael Traugott

**Affiliations:** ^1^ Institute of EcologyUniversity of InnsbruckTechnikerstraße 256020InnsbruckAustria

**Keywords:** gut content analysis, *Phalacrocorax carbo sinensis*, piscivorous birds, prey identification, secondary predation

## Abstract

Avian carcasses can provide important information on the trophic ecology of birds. Usually, the number of carcasses available for examination is limited and therefore it is important to gain as much dietary information per specimen as possible. In piscivorous birds and raptors, the stomach has been the primary source of dietary information, whereas the gut (intestine) has so far been neglected as it usually contains only a few morphologically identifiable hard parts of prey. Molecular approaches have the potential to retrieve dietary information from the gut, although this has not yet been verified. As well as identifying the prey, it is important to estimate any secondary predation to avoid food web errors in dietary analyses. The assignment of accidentally consumed prey is notoriously difficult regardless of the prey identification approach used. In the present study, morphological and molecular analyses were, for the first time, combined to maximize the dietary information retrievable from the complete digestive tract of Great Cormorants *Phalacrocorax carbo sinensis*. Moreover, a novel approach based on predator–prey size ratios was applied to these piscivorous birds to minimize the number of samples that might contain secondarily predated prey. The stomach contents of the examined birds were found to provide the most dietary information when morphological and molecular analyses were used in combination. However, compared with the morphological approach, the molecular analysis increased the number of fish species detected by 39%. The molecular approach also permitted the identification of fish DNA in the Cormorant guts. Predator–prey size ratios derived from morphological analysis of fish hard parts can reduce the incidence of potential confounding influence of secondarily predated prey by 80%. Our findings demonstrate that a combination of morphological and molecular approaches maximizes the trophic information retrievable from bird carcasses.

Examining diet and assessing how it relates to evolutionary and environmental processes is central to understanding the biology and ecology of birds. For example, knowing which food sources sustain birds is vital to protecting endangered species (e.g. Li *et al*. 2014), to managing birds species that either compete for prey with humans (e.g. Ostman *et al*. 2013) or reduce vertebrate and invertebrate pests (Paz *et al*. 2013, Flower *et al*. 2014) and more broadly to understanding the role that birds play in terrestrial and aquatic food webs (e.g. Mantyla *et al*. 2011, Green & Elmberg 2014).

The examination of the digestive tracts of dead specimens is common practice in avian dietary studies. Digestive tracts can provide a valuable source of dietary information, including undigested whole prey remains and indigestible prey hard parts, as well as macerated, semi‐digested prey. Molecular techniques can be used to identify prey within the latter sample type and represent an important technical advance, as analysis of pre‐digested pulpy material usually contains no morphologically identifiable prey remains. In contrast, whole prey specimens and undigested bones can often be identified to a lower taxonomic level using specific morphological features. Prey hard parts also offer the opportunity reliably to estimate prey size and mass (e.g. Gagliardi *et al*. 2007, März 2007).

Although the examination of indigestible hard parts works well for vertebrate prey, usually no prey remains can be retrieved from soft‐bodied and small prey, such as fish fry, small fish and invertebrates (e.g. Barrett *et al*. 2007, März 2007). Furthermore, hard part recovery rates can vary with size and between prey species (e.g. Zijlstra & Vaneerden 1995, McKay *et al*. 2003) and it can be difficult to identify prey remains because they are strongly eroded by digestion and broken (e.g. Gagliardi *et al*. 2007, März 2007) or because closely related taxa cannot be distinguished (e.g. Veldkamp 1995a, Suter 1997, Keller 1998, Klein & Lieser 2005, Stewart *et al*. 2005, März 2007). Molecular techniques can overcome many of these limitations inherent to morphological hard part analysis, as soft and semi‐digested gut content as well as ambiguous prey hard parts can be identified (e.g. Alonso *et al*. 2014, Egeter *et al*. 2015). However, the high sensitivity of molecular detection systems bears the risk of detecting DNA of prey that was ingested via secondary predation (Sheppard *et al*. 2005). Secondary predation occurs when a secondary predator such as a bird consumes a primary predatory species that in turn was feeding on prey that might be mistakenly assigned as being preyed upon by the secondary predator, leading to erroneous food web construction. Here, we propose the novel idea that the relationship between the size of the hard part prey remains of both the putative prey and the primary predator can be used to assess whether the prey is likely to have been consumed by the primary predator. If the prey is small enough to be consumed by the primary predator, secondary predation by birds is a possibility, but if the prey is found to be too large to have been consumed by the primary predator, secondary predation can be ruled out. The rationale of this approach is that consumers are usually larger than their prey species (Brose *et al*. 2006). So if one can estimate the size of the prey species found in a bird's gut sample, the predator–prey size ratios can be calculated. This in turn enables determination of those predator–prey combinations that are unlikely to have occurred due to the prey being too large for the putative primary predator to have consumed.

Most previous dietary examinations of dead birds have used either molecular or morphological prey identification methods; a combination of the two approaches could compensate for their respective methodological weaknesses and result in a more detailed picture of a predator's prey choice (Casper *et al*. 2007, Deagle *et al*. 2007, Tollit *et al*. 2009, Braley *et al*. 2010). For example, in a study of Cory's Shearwater *Calonectris borealis*, a combination of molecular and morphological approaches improved prey identification and quantification in stomach content samples (Alonso *et al*. 2014). However, the extent to which parts of the digestive tract other than the stomach also contain food remains that could be identified in dietary analyses using both molecular and morphological approaches remains untested.

Here, we assess whether the combination of morphological and molecular analysis improves the dietary information that can be retrieved not only from stomachs but also from different parts of the gut. We predicted that combining morphological and molecular techniques would reveal a wider and more detailed prey spectrum than when using a single approach, and that molecular analysis would be especially useful in detecting prey in different parts of the digestive tract. These expectations were tested by examining the gut contents of carcasses of Great Cormorants *Phalacrocorax carbo,* a fish‐eating bird occurring throughout the northern hemisphere. As Cormorants are viewed as competitors to fisheries, these birds are legally shot in many countries to reduce their population densities and their impact on fish populations (e.g. Harris *et al*. 2008, Vetemaa *et al*. 2010, Marzano & Carss 2012). Hence, carcasses of Cormorants are available in high numbers for dietary analyses. The Cormorant carcasses examined in this study were also used to test the predator–prey size estimation from prey hard parts to assess the probability of secondary predation.

## Methods

### Origin of Cormorants and sample preparation

The 35 Cormorants analysed in this study were obtained from the vicinity of the fish farm of the Bavarian Environment Agency (LfU) in Wielenbach, Bavaria, Germany: 23 individuals were shot on the River Lech and 12 individuals were shot at ponds in the fish farm. At the time of the cull, the following fish species were farmed in the ponds: Carp *Cyprinus carassius*, Grass Carp *Ctenopharyngodon idella*, Tench *Tinca tinca*, Asp *Aspius aspius*, Pike *Esox lucius*, Burbot *Lota lota* and Pikeperch *Sander lucioperca*. The Cormorants were shot during the winter migration period between October 2011 and April 2013 as a measure to control and scare off overwintering Cormorants from those sites, with all required permissions. After being shot, the birds were collected within 5 days, depending on how long it took for them to be washed ashore. Their carcasses were frozen and brought to the University of Innsbruck, where they were stored at −28 °C. For further analyses the birds were thawed and dissected, and the stomach and the gut were removed and unravelled carefully. Thereafter, the digestive tract of each bird was divided into the stomach and the gut, which was separated into three gut parts of equal length.

**Figure 1:**
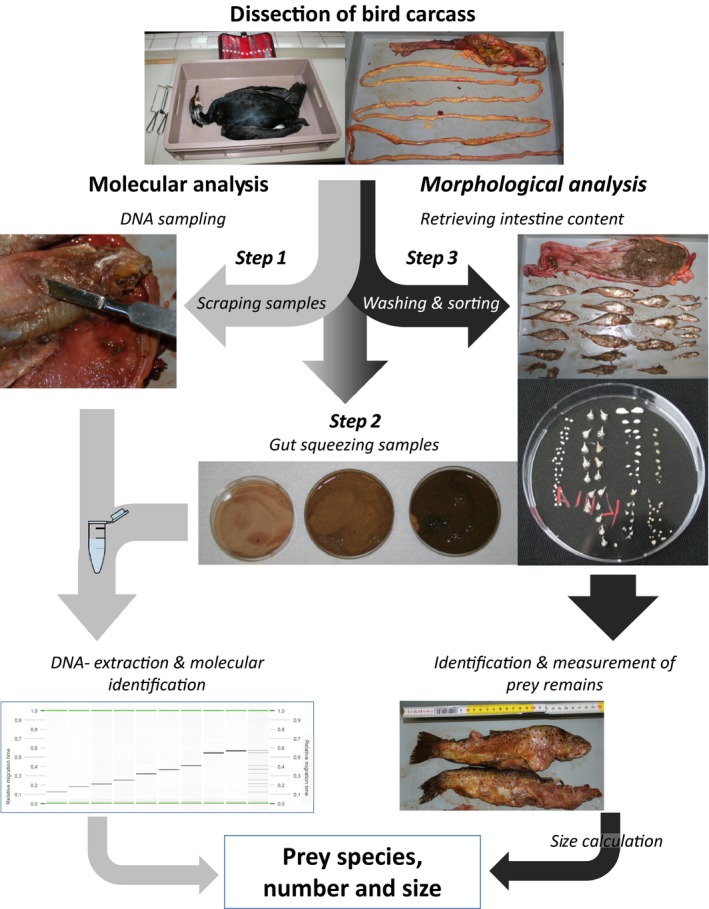
Workflow of sample preparation, from the carcass to the results of morphological and molecular gut content analyses. Grey arrows represent molecular and black arrows the morphological procedures illustrating how the trophic data were generated.

Each stomach was cut open and scraping samples of the undigested fish and of the gastric mucosa were taken to obtain prey DNA potentially present on undigested fish remains and on the gastric mucosa of the stomach, respectively. Approximately 0.5 mL of the recovered tissue was transferred into 1.5‐mL reaction tubes (step 1 in Fig. 1). The liquid contents of the three gut parts were squeezed separately into Petri dishes (10 cm diameter), mixed by stirring with a DNA‐free spatula and then approximately 0.5 mL was transferred into a 1.5‐mL reaction tube (step 2 in Fig. 1). All samples were frozen at −28 °C for later molecular analysis.

### Molecular analysis

The molecular work generally followed the protocol outlined in Thalinger *et al*. (2015) with some modifications. For tissue lysis, all samples were defrosted and 1 mL buffer solution (TES‐buffer and proteinase K (10 mg/mL); 43 : 1, v/v) was pipetted into each reaction tube. The samples were vortexed and placed into an incubator at 56 °C for 24 h. Subsequently, the BioSprint 96 robotic platform (Qiagen, Hilden, Germany) was used for DNA extraction using the BioSprint 96 DNA Blood Kit (Qiagen) in accordance with the manufacturer's instructions. For each 96‐well plate, 92 lysates and four extraction negatives (TES‐buffer instead of lysate) were processed. The extracted DNA, recovered in 200 μL TE elution buffer, was transferred to 2‐mL reaction tubes and stored at −32 °C.

The DNA extracts were screened for fish DNA using the multiplex PCR systems for European freshwater fish (Thalinger *et al*. 2015). This multiplex PCR system consists of six molecular assays targeting the mitochondrial 16S rRNA (16S) and the cytochrome *c* oxidase subunit I (COI) genes, which enables the molecular identification of all prey fish species potentially consumed by the Cormorants. All samples which tested negative after a first screening with the family‐specific multiplex PCR assay (‘FishTax’) were spiked with ~50 ng of Perch *Perca fluviatilis* DNA and retested to check for the presence of PCR inhibitors.

### Morphological analysis

After dissection, the contents of each of the three gut parts were sieved separately and rinsed with tap water to remove soft parts and to make the prey hard parts visible. Undigested fish and fish parts in the stomach were identified morphologically and their length was measured. Thereafter, the stomach material was also sieved, rinsed with water, and inspected for additional prey hard parts (step 3 in Fig. 1). Hard parts such as otoliths, pharyngeal teeth, chewing pads and jaws were sorted out for prey identification. These fish remains were identified using the identification keys of Härkönen (1986) and by comparing them with fish bone reference collections provided by Dr Werner Suter (Swiss Federal Research Institute, Birmensdorf, Switzerland), Dr Josef Trauttmansdorff (Otto‐Koenig Institute, Stockerau, Austria) and the Bavarian State Collection of Zoology (Munich, Germany). The sagittae (large otolith) were used for identification of non‐cyprinids, and pharyngeal bones, pharyngeal teeth and chewing pads were used for cyprinid identification. In addition, hard parts such as lenses and chewing pads were used to estimate the number of individual fish present in the gut.

The length of the fish prey was either measured directly on undigested fish or calculated from fish‐bone regressions. For the latter, intact key bones were measured under a dissecting microscope to the nearest 0.1 mm and fish lengths were calculated using regression formulae for sagittae (Härkönen 1986, Emmrich & Duettmann 2011, Gaye‐Siessegger 2014), chewing pads (Veldkamp 1995b, Emmrich & Duettmann 2011), and pharyngeal bones, jaws and praeopercula (Cech 2006, Emmrich & Duettmann 2011).

To estimate the probability of secondary predation, the stomachs that contained predatory fish as well as their potential prey fish species were selected. In stomachs in which undigested predatory fish and their potential prey were present next to each other, secondary predation was ruled out. For the remaining cases only the hard parts of predatory fish and their potential fish prey were present. The minimum and maximum sizes of these fish were calculated using the regression formulae described previously. Within each stomach sample and for each predator–prey species combination, the minimum and maximum size ratio between the putative prey and predator was calculated. In each case, where the estimated size of the putative prey fish was smaller than 51% of the potential piscivorous fish, secondary predation was deemed possible (Scharf *et al*. 2000, Dörner & Wagner 2003).

### Data analysis

Wilcoxon signed ranks tests were used to compare prey detection rates and the mean number of prey species detected between data generated morphologically and molecularly (non‐parametric paired data; *P*‐value reported only). The mean numbers of prey species detected morphologically and molecularly in the different sections of the digestive tract of all birds (unpaired data) were compared using Mann–Whitney *U*‐tests (*U‐* and *P*‐values reported) as well as one‐sample *t*‐tests combined with bootstrapping for 9999 resamples to generate 95% tilting confidence intervals (*TCIs*). All tests were performed with spss 12.1 (SPSS Inc., Chicago, IL, USA), except the calculation of the TCIs, for which s‐plus 8.0 (Insightful Corporations, Seattle, WA, USA) was used.

## Results

### Prey detection using morphological and molecular analyses

**Figure 2:**
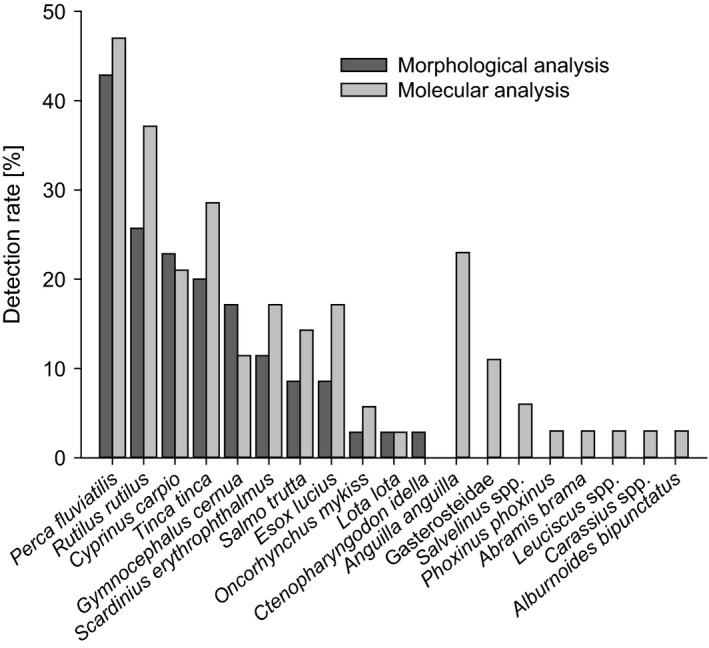
Fish species detected in the digestive tracts of dead Cormorants (*n *=* *35) and their respective detection rate per bird using morphological or molecular analysis.

In three (9%) of the 35 Cormorant stomachs surveyed, no prey hard or soft parts were recovered. In contrast, all guts contained pre‐digested soft material. Only one gut sample contained fish hard parts (eye lens and an unidentifiable otolith), so prey identification based on morphological hard part analysis was only possible for stomach samples. The morphological analysis of the hard parts allowed for the identification of 336 individual fish of 11 species (Fig. 2). Every stomach with visible prey remains contained hard parts of prey that allowed calculation of prey number and size. The average number of individual fish found per stomach was 11 ± 15 sd (range 1–60) with a mean prey length of 107 ± 62 mm sd, ranging from 35 mm in *Perca fluviatilis* to 356 mm in *Oncorhynchus mykiss*.

Molecular analysis resulted in the identification of 18 fish species (Fig. 2). Most of the fish species additionally detected by molecular means were present in only one bird, shot on the River Lech, whereas DNA of *Anguilla anguilla* was amplified in 25% of the gut samples even though morphologically discernible prey remains of this species were not recovered in any of the samples. Furthermore, in all but one of the stomach samples in which no prey hard parts were recovered, fish DNA was detected. Altogether, fish DNA could be PCR‐amplified in 59% of the gut samples. The DNA extracts of those samples that did not test positive in the multiplex PCR system tested positive with the FishTax assay when spiked with DNA of *P. fluviatilis*, suggesting that PCR inhibition was not a factor in our analyses of diet.

**Figure 3:**
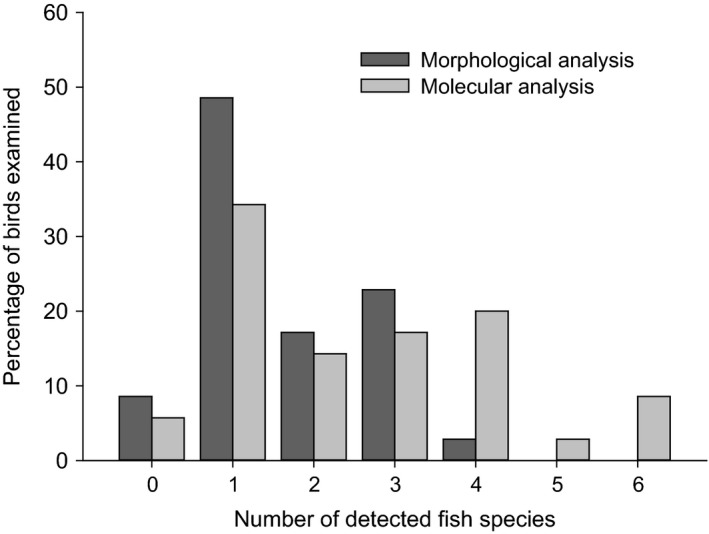
Maximum number of fish species detected in Cormorant carcasses (*n *=* *35) examined using morphological or molecular methodology.

In bird stomachs, significantly more fish species were detected molecularly (mean: 2.6 ± 1.7 sd species; maximum six species per stomach) than morphologically (mean: 1.6 ± 1.0 sd species; maximum four species per stomach; Fig. 3, Wilcoxon *P *<* *0.001). In two stomach samples the molecular methodology permitted identification of the prey fish to species level, whereas with the morphological approach taxonomic identification was possible to family level only.

**Figure 4:**
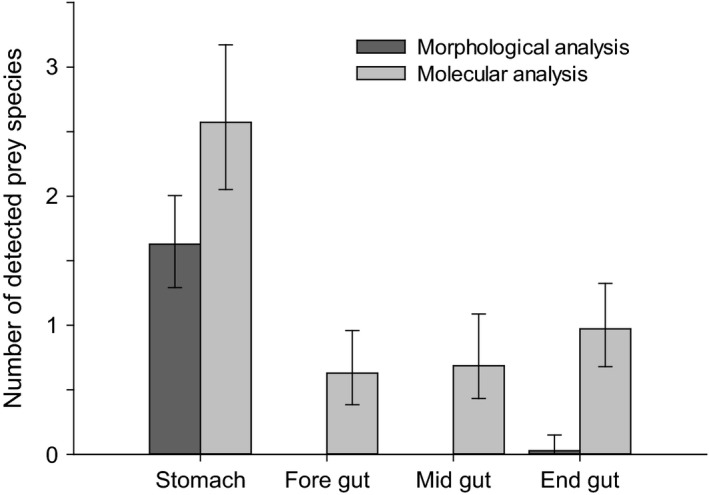
Mean number of prey species detected (±95% tilting confidence intervals) morphologically and molecularly in different digestive tract sections of the Cormorant carcasses (*n *=* *35).

In none of the birds did either the morphological or the molecular prey detection approach permit identification of a prey species in the gut sections that was not already detected in the corresponding stomach sample. The number of detected prey species decreased significantly from stomach to the first gut section in samples analysed both morphologically (from mean 1.6 ± 1 sd to 0 species) and molecularly (from mean 2.6 ± 1.7 to 0.6 ± 0.8 species; *U *=* *170, *P *<* *0.001; Fig. 4). No significant differences occurred in the average number of prey species detected between the first and second gut sections (morphological analysis: 0 species; molecular analysis: from mean 0.6 ± 0.8 sd to 0.7 ± 0.9 sd species, *U *=* *589.5, *P *=* *0.765), or between second and third gut sections (morphological analysis: 0 species; molecular analysis: from mean 0.7 ± 0.9 sd to 1.0 ± 0.9 sd species, *U *=* *498.5, *P *=* *0.148; Fig. 4).

### Estimating the levels of secondary predation

The DNA of piscivorous fish species (*A. anguilla*,* P. fluviatilis*,* Esox lucius*,* O*. *mykiss* and *Salmo trutta*) was detected in 23 of the 35 dissected Cormorants, with 19 gut samples containing DNA of piscivorous fish and their potential prey. Likewise, hard parts of piscivorous fish were found in 22 of the Cormorants examined, with 12 stomachs also containing remains of their potential fish prey. These remains were used to estimate the size relationship between the putative predator and prey: on average the putative prey fish was 60 ± 35% sd the size of the potential predatory fish. In 28 of the 35 stomach samples, secondary predation could be excluded because either no potential prey was detected next to the putative predator or the potential prey size has a ratio of greater than 51% of its putative predator length. Dörner and Wagner (2003) found 51% to be the maximum predator–prey size ratio for Perch when feeding on Perch and Roach and therefore this value was taken as a threshold to estimate the probability of secondary predation.

## Discussion

### Prey detection in different parts of the digestive tract

For only one of the 35 Cormorants analysed were fish hard parts recovered in the gut. This is not surprising as indigestible prey remains are usually discarded by regurgitation in carnivorous bird species (Barrett *et al*. 2007, März 2007) so very few hard parts enter the gut and are defecated (Johnson & Ross 1996). Therefore, the gut content and faeces cannot be used for comprehensive morphological diet analysis of these birds. Nevertheless, the gut content and faeces can provide valuable dietary information when molecular methods are applied, even when the stomach contains no visible hard parts. On average, 61% of the fish species detected in the stomach were also detected in at least one of the gut sections. However, no prey DNA was detected in 41% of the gut samples. Possibly there was a lack of amplifiable prey DNA present in the gut because of prey DNA breakdown. The Cormorants were collected up to 5 days after death, so DNA decay caused by microbial and enzymatic activity in the digestive tract of the birds is likely to have started. It has also been suggested that substances that can inhibit PCR can be produced within decomposing faecal material (Deagle *et al*. 2005). This can also apply for the gut contents because the material is already pre‐digested and PCR inhibitors could be produced by microbes. Inhibition, however, does not seem to have caused the lack of PCR‐amplification in the negatively tested Cormorant gut samples, as amplification of molecular markers was successful when spiked with Perch DNA. This indicates that these samples were genuinely lacking prey DNA and thus true negatives.

### Prey species detection and prey size

Our study shows that the addition of molecular tools to the morphological analysis of prey remains increases the prey spectrum detected in the digestive tracts of Great Cormorants by 39%. This confirms results of previous studies on piscivores: for example, Tollit *et al*. (2009) detected in faeces of Steller's Sea Lions *Eumetopias jubatus* DNA of four fish species that left no hard parts in the dietary samples, and Braley *et al*. (2010) detected 33% more prey species molecularly than morphologically in the stomachs of Arrow Squids *Nototodarus gouldi*. Casper *et al*. (2007) found no dietary information in 25.9% of faecal samples of Antarctic Fur Seals *Arctocephalus gazella* when applying either the morphological or the molecular approach, but this was reduced to 9.3% when combining the two methods.

The reason that certain fish species were not detectable by prey hard part remains in the present study is either because they are very small and therefore get easily digested (e.g. *Phoxinus phoxinus*,* Gasterosteus* spp.) or because the hard parts only permit identification to the family level. The molecular analyses help to overcome these shortcomings, allowing a more complete prey spectrum to be assessed. Even in two stomach samples, in which no visible prey content was found, the molecular analysis detected fish DNA. This is surprising because these birds must have digested their most recent prey and regurgitated its hard parts along with the Cormorant stomach mucosa (Trauttmansdorff & Wassermann 1995, Zijlstra & Vaneerden 1995), which should leave no or very little fish DNA to be detected. The PCR‐amplification of fish DNA from these samples indicates the high sensitivity of the multiplex PCR assays applied in our study, in which 25 template molecules per reaction were sufficient to detect the prey (Thalinger *et al*. 2015). However, the high sensitivity of these assays also carries the risk of false positive prey detection: DNA of non‐ingested fish species might stick to the consumed fish, as both fish types occur in the same environment (Rees *et al*. 2014). Detection of such environmental DNA (eDNA) of fish in the context of assessing trophic interactions could lead to food web errors and an artificially inflated prey spectrum of the consumer species. However, compared with the amount of DNA from truly ingested prey, the copy number of eDNA should be very small. Additionally, eDNA rapidly disintegrates into small fragments in the water (Rees *et al*. 2014) and thus it is likely to be highly fragmented when ingested by piscivorous birds. Moreover, eDNA sticking to the body surface of the predated fish is probably digested quickly, as it is only present in very small amounts and directly exposed to digestion enzymes. We think that it is unlikely that eDNA has biased the current findings due to the reasoning described above, but it seems warranted for future studies to assess whether and how strongly the presence of eDNA can affect the outcome of molecular dietary studies.

Molecular methods enable prey detection at high specificity and sensitivity but quantification of ingested food remains difficult (King *et al*. 2008, Deagle *et al*. 2013). At best, the proportion of different prey species can be estimated based on prey DNA concentrations or number of prey sequences: this approach has been applied in diet analyses of perches (Taguchi *et al*. 2014), pinnipeds (e.g. Deagle & Tollit 2007, Bowles *et al*. 2011, Deagle *et al*. 2013) and penguins (Deagle *et al*. 2010) and correction factors have been generated to improve diet estimates based on sequence proportions (Thomas *et al*. 2014). In contrast to molecular techniques, prey hard parts offer a straightforward way to estimate the number and size of prey individuals. Although this approach also has its shortcomings, as otoliths can be missing (Johnstone *et al*. 1990) or reduced in size due to digestive erosion, prey hard part remains were measured in this study to estimate the size of the fish via regression formulae available for a wide variety of fish taxa (e.g. Härkönen 1986, Gaye‐Siessegger 2014).

### Secondary predation

In the present study, secondary predation of fish could be excluded in 80% of the Cormorants based on the size ratio between the putative predator and prey calculated from their hard part prey remains. This allowed us significantly to reduce the number of instances in which secondary predation could lead to food web errors in our dataset. Our results confirm the results of Blackwell and Sinclair (1995) on Double‐crested Cormorants *Phalacrocorax auritus*, who found no significant differences in the size of otoliths recovered from regurgitated fish and from fish stomachs of regurgitated fish. To our knowledge there is no methodology that could resolve whether the remaining questionable cases contain primary or secondary prey for either the morphological or molecularly generated data. One way to deal with this problem is either to assume that all of the prey detected in these samples has been directly consumed, or to assume the opposite, i.e. all potential secondarily consumed prey was indirectly predated. Checking whether and how these opposing assumptions affect the trophic analysis would help determine whether secondary predation is an issue that needs to be considered for data interpretation. Secondary predation can occur in piscivorous birds and raptors feeding on carnivorous and insectivorous mammals and birds. For example, März (2007) found that insect remains in raptors originate from the craw of predated birds. Estimating the probability of secondary predation using predator–prey size ratios, as outlined in this study, would be widely applicable for studies dealing with vertebrate prey, provided that the predator consumed the respective hard parts.

This study demonstrates that molecular techniques offer the possibility of detecting prey in avian carcasses when their stomachs are empty, a frequent occurrence when examining field‐collected birds (Ouwehand *et al*. 2004, Stewart *et al*. 2005). Molecular analysis also yields a wider prey spectrum than morphological analysis of prey remains. Morphological prey remains are vital for estimating prey number and size. Furthermore, the latter enables us to estimate the possibility of secondary predation based on predator–prey size ratios and to reduce the instances of potential secondarily predated prey. Beyond the combination of molecular and morphological approaches, the dietary information retrievable from carcasses of birds could be extended further by including the analysis of stable isotopes and fatty acids (Inger & Bearhop 2008, Williams & Buck 2010); these approaches would enable an assessment of the long‐term diet at a more general level (Traugott *et al*. 2013) compared with the time‐specific diet information retrievable from the digestive tract.

This study was funded by the Austrian Science Fund (FWF), project number P24059, entitled ‘The feeding ecology of the Great Cormorant’. We thank Matthias Ruff from the state fish farm Wielenbach (Germany) for providing the Cormorants, Lena Manzl for assisting with the dissections and the members of the Applied and Trophic Ecology research group as well as two anonymous reviewers, the Associate Editor and Rauri Bowie for valuable discussions and suggestions on the manuscript.
